# Dysregulated HAI-2 Plays an Important Role in Renal Cell Carcinoma Bone Metastasis through Ligand-Dependent MET Phosphorylation

**DOI:** 10.3390/cancers10060190

**Published:** 2018-06-08

**Authors:** Koji Yamasaki, Shoichiro Mukai, Satoru Sugie, Takahiro Nagai, Kozue Nakahara, Toyoharu Kamibeppu, Hiromasa Sakamoto, Noboru Shibasaki, Naoki Terada, Yoshinobu Toda, Hiroaki Kataoka, Toshiyuki Kamoto

**Affiliations:** 1 Department of Urology, Faculty of Medicine, University of Miyazaki, Miyazaki 889-1692, Japan; koji_yamasaki@med.miyazaki-u.ac.jp (K.Y.); satoru_sugie@med.miyazaki-u.ac.jp (S.S.); takahiro_nagai@med.miyazaki-u.ac.jp (T.N.); kozue_nakahara@med.miyazaki-u.ac.jp (K.N.); toyoharu@med.miyazaki-u.ac.jp (T.K.); naoki_terada@med.miyazaki-u.ac.jp (N.T.); tkampro@med.miyazaki-u.ac.jp (T.K.); 2 Department of Urology, Faculty of Medicine, University of Kyoto, Kyoto 606-8507, Japan; sakamoto@kuhp.kyoto-u.ac.jp (H.S.); noboru@kuhp.kyoto-u.ac.jp (N.S.); 3 Department of Clinical Laboratory Science, Tenri Health Care University, Nara 632-0018, Japan; todayadot@gmail.com; 4 Oncopathology and Regenerative Biology Section, Faculty of Medicine, University of Miyazaki, Miyazaki 889-1692, Japan; mejina@med.miyazaki-u.ac.jp

**Keywords:** RCC, bone metastasis, HGF, MET, HAI-2

## Abstract

MET, a *c-met* proto-oncogene product and hepatocyte growth factor (HGF) receptor, is known to play an important role in cancer progression, including bone metastasis. In a previous study, we reported increased expression of MET and matriptase, a novel activator of HGF, in bone metastasis. In this study, we employed a mouse model of renal cell carcinoma (RCC) bone metastasis to clarify the significance of the HGF/MET signaling axis and the regulator of HGF activator inhibitor type-2 (HAI-2). Luciferase-transfected 786-O cells were injected into the left cardiac ventricle of mice to prepare the mouse model of bone metastasis. The formation of bone metastasis was confirmed by whole-body bioluminescent imaging, and specimens were extracted. Expression of HGF/MET-related molecules was analyzed. Based on the results, we produced HAI-2 stable knockdown 786-O cells, and analyzed invasiveness and motility. Expression of HGF and matriptase was increased in bone metastasis compared with the control, while that of HAI-2 was decreased. Furthermore, we confirmed increased phosphorylation of MET in bone metastasis. The expression of matriptase was upregulated, and both invasiveness and motility were increased significantly by knockdown of HAI-2. The significance of ligand-dependent MET activation in RCC bone metastasis is considered, and HAI-2 may be an important regulator in this system.

## 1. Introduction

Renal cell carcinoma (RCC) is the most common type of kidney malignancy in adults [1]. Approximately 20–30% of RCC patients are reported to have metastasis at the time of diagnosis [1], and 30% of advanced RCC patients revealed bone metastasis in modern randomized clinical trials [2,3]. There is a correlation between bone metastasis and poor prognosis, and osteolytic metastasis significantly affects patient quality of life through skeletal-related events (SREs) [4,5]. The clinical application of molecular targeted drugs such as vascular endothelial growth factor receptor (VEGFR), targeted tyrosine kinase inhibitor (TKI), and mammalian target of rapamycin (mTOR) inhibitors have improved the treatment of metastatic RCC (mRCC). The overall survival benefit of sunitinib is estimated at five months compared with interferon α, and axitinib has clinical benefit in patients with failed response to prior molecular targeted drugs [6]. Although these have advanced the treatment of mRCC, complete remission and long-term survival remain rare. Recent advances in this field have led to treatment with new agents including nivolumab and cabozantinib in second- or third-line settings, and each agent has demonstrated favorable efficacy [7,8,9,10]. Cabozantinib is a potent inhibitor of MET, a *c-met* proto-oncogene product and receptor for hepatocyte growth factor (HGF) and VEGFR-2. MET is known to play an important role in the progression of various cancers, including RCC through activation by an active form of HGF [7,8,9,10], and high MET expression with worsening prognosis has been reported [11,12,13,14,15]. Activation of MET can lead to the activation of several cell signaling pathways, including Src kinase, the phosphatidylinositol 3-kinase (PI3)/AKT/mTOR pathway and the Ras/Raf/MEK/ERK (ordinary MAP kinase) pathway [11,12,14,15,16]. In cancer cells, activation of these signaling pathways is reported to promote cell proliferation, survival (anti-apoptosis), motility, invasiveness, and epithelial-mesenchymal transition (EMT) [12,16]. In our previous study, higher expression of MET and matriptase was observed in bone metastasis compared with nephrectomy specimens by immunohistochemistry [14]. Furthermore, the overall postoperative survival rate was significantly higher in the MET negative group than in the MET positive group, which indicates that MET and matriptase affect not only the formation of bone metastasis, but also patient survival. However, the roles of those factors in bone metastasis have not yet been clarified. Matriptase is a type-2 transmembrane serine protease that is known as a sufficient activator of hepatocyte growth factor zymogen (pro-HGF) [12,17,18]. Elevated matriptase expression is significantly associated with tumor aggressiveness through activated HGF-induced phosphorylation of MET [12,17,19,20,21,22]. On the other hand, matriptase is negatively regulated by HGF activator inhibitor type-2 (HAI-2), a Kunitz-type serine proteinase inhibitor, and has a correlation with matriptase in the regulation of tumor migration, invasion and metastasis [23].

In this study, we employed a mouse model of bone metastasis to clarify the significance of the MET signaling pathway in RCC bone metastasis. As a result, we considered the importance of the HGF/MET signaling axis through the HAI-2-induced regulation of pro-HGF activation in bone metastasis. In addition, we examined the biological function of HAI-2 in RCC cells using both HAI-2 stable knockdown and engineered expression RCC cells.

## 2. Results

### 2.1. Expression of Each Molecule in RCC Cell Lines

**Figure 1:**
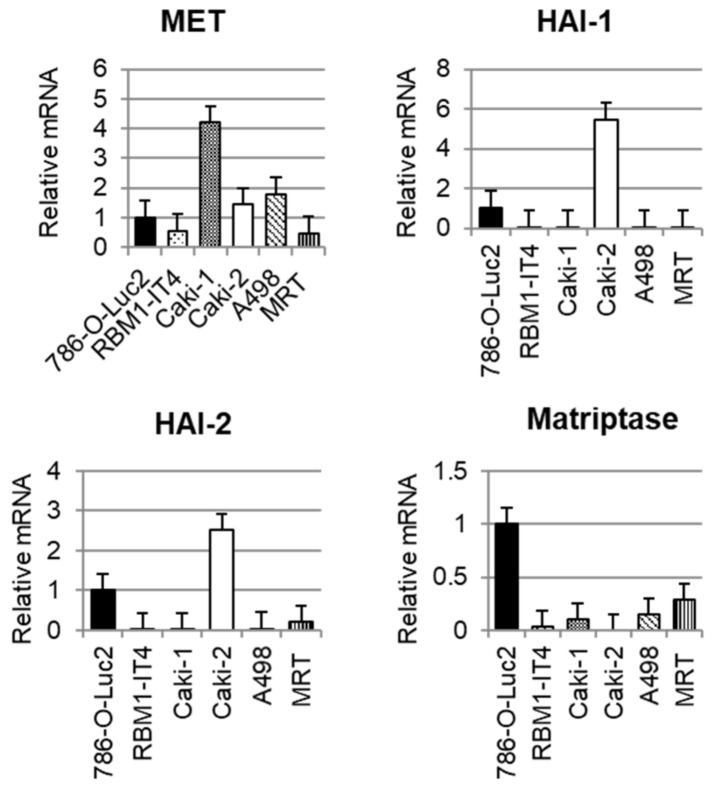
RT-qPCR analyses of MET, a *c-met* proto-oncogene product and receptor for hepatocyte growth factor (HGF), HGF activator inhibitor-1 (HAI-1), and HAI-2 and matriptase in six renal cell carcinoma cell lines. Glyceraldehyde-3-phosphate dehydrogenase (GAPDH) mRNA was used as the internal control.

We examined the expression of MET, HAI-1, HAI-2, and matriptase mRNA in RCC cell lines using real-time quantitative PCR (RT-qPCR). As shown in Figure 1, all cell lines expressed MET. Expression of matriptase was observed in Luciferase transfected 786-O (786-O-Luc2) cells. HAI-1 expression was increased in Caki-2, and HAI-2 was expressed in Caki-2 and 786-O-Luc2 cells; however, the expression of HAIs was downregulated in other cell lines, including bone metastasis-derived cell line RBM1-IT4.

### 2.2. Preparation of Mouse Model of Bone Metastasis

**Figure 2:**
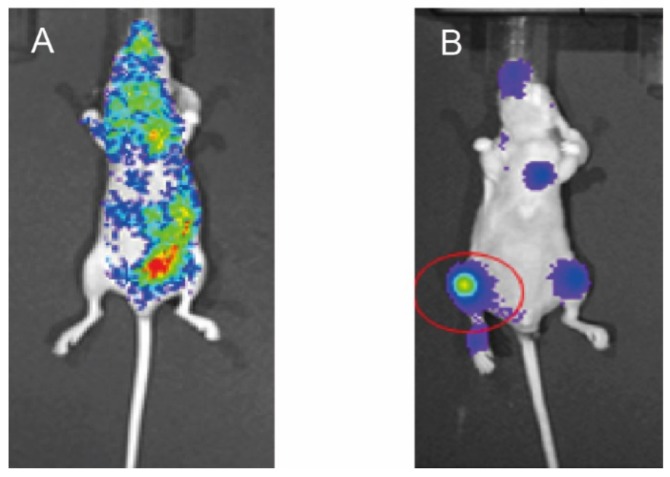

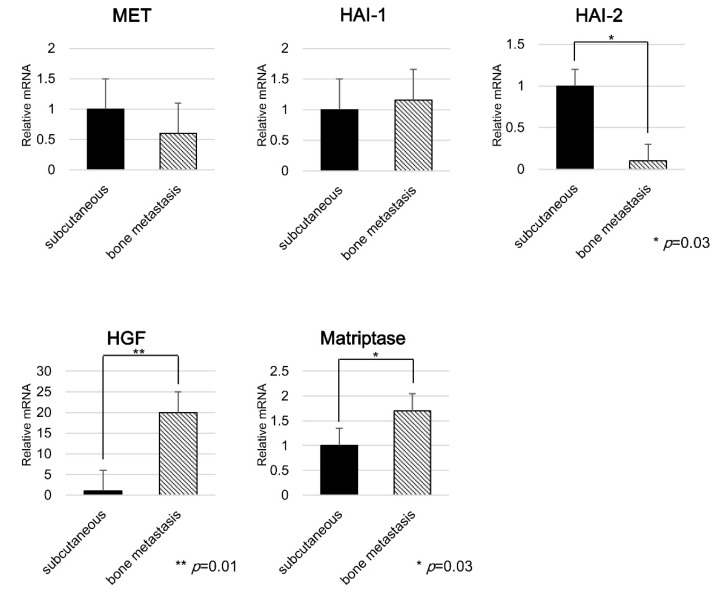
Bioluminescent imaging of the mouse model. After intra-cardiac injection (**A**), six weeks from injection (**B**). Apparent bone metastasis is confirmed (**B**), red circle. Extracted specimens were used for RT-qPCR analyses. mRNA expression of MET, HAI-1, HAI-2, HGF and matriptase in bone metastasis and subcutaneous implantation of 786-O-Luc2 cells (**C**).

We employed a mouse model of RCC bone metastasis. 786-O-Luc2 cells were injected into the left cardiac ventricle of 5-week-old mice. Whole body distribution of 786-O-Luc2 was observed by bioluminescent imaging in three successfully injected mice (Figure 2A). Bone metastasis formation was confirmed six weeks after injection in all three mice by the same procedure (Figure 2B), and then metastatic tissue was extracted (metastasis at femoral bone, red circle). In addition, subcutaneously implanted 786-O-Luc2 cells were extracted from tissue samples obtained from three other mice after sacrifice at six weeks from implantation for use as control.

### 2.3. Expression of Each Molecule in Bone Metastasis

**Figure 3:**
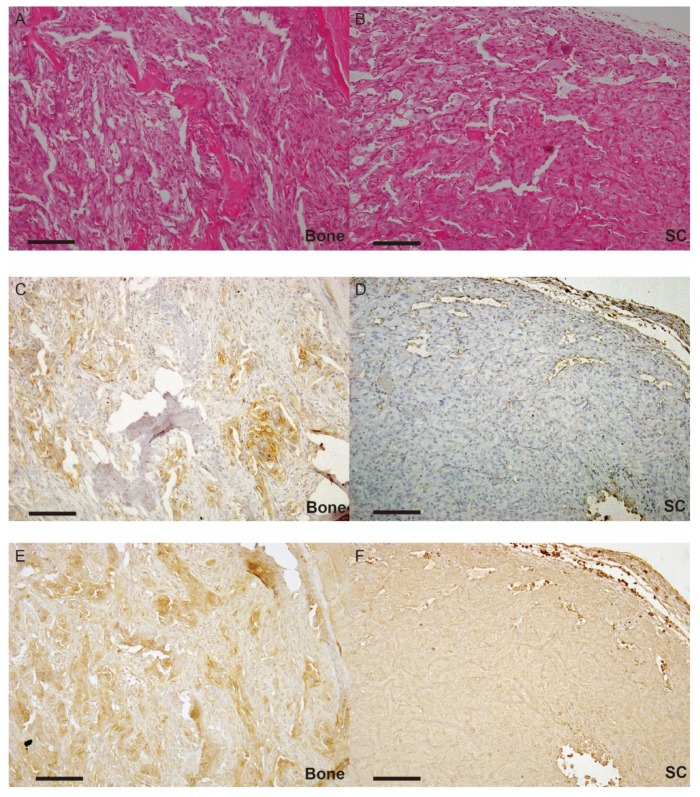
Pathological and immunohisitochemical appearance of bone metastasis and subcutaneous implantation of 786-O-Luc2 cells using serial tissue sections. (**A**) Bone metastasis (bone), stained with hematoxylin and eosin (H&E); (**B**) subcutaneous implantation (SC), stained with H&E; (**C**) bone, phosphorylation of MET (p-MET) immunostaining; (**D**) SC, p-MET immunostaining; (**E**) bone, total-MET (t-MET) immunostaining; (**F**) SC, t-MET immunostaining. Scale bars = 100 μm.

Extracted tissues from bone metastasis were analyzed, and subcutaneously implanted specimens were used as control. RT-qPCR revealed that mRNA expression of matriptase and HGF increased in bone metastasis, while that of HAI-2 was significantly decreased. Immunohistochemical analysis revealed significantly enhanced phosphorylation of MET in bone metastasis compared with subcutaneous implanted tumor cells (Figure 3B,E). These results suggested the significance of ligand-dependent MET activation in an autocrine manner with this model. MET was diffusely expressed in cancer cells from both implanted subcutaneous tissue and bone (Figure 3C,F). Interestingly, the proportion of spindle-shaped cancer cells increased in bone metastasis compared with subcutaneously implanted tumor cells in hematoxylin eosin staining (Figure 3A,D).

### 2.4. In Vitro Study Using HAI-2 Stable Knockdown and Overexpressed RCC Cells

**Figure 4:**
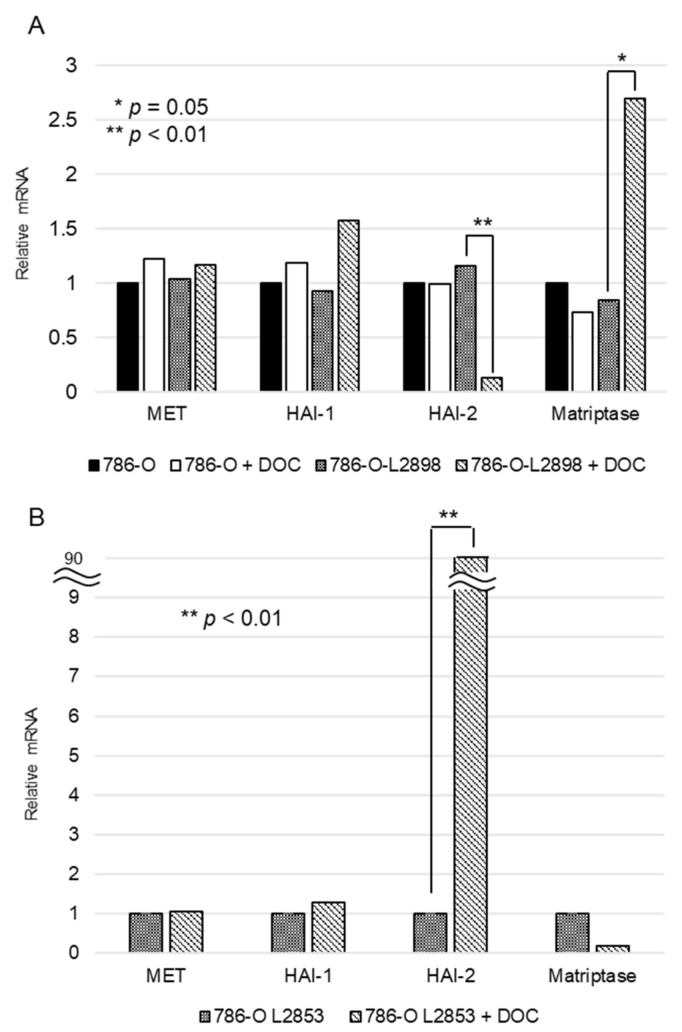
(**A**) Expression of HAI-2 was confirmed by RT-qPCR analyses in HAI-2-knockdown 786-O-Luc2 (786-O-L2898) with and without 0.5 μg/mL of doxycycline (DOC). Effect of HAI-2-knockdown on expression of MET, HAI-1 and matriptase was also verified; (**B**) Expression of HAI-2, MET, HAI-1 and matriptase were confirmed in HAI-2-engineered expressed 786-O-Luc2 (786-O-L2853) cells with and without 0.5 μg/mL of doxycycline.

**Figure 5:**
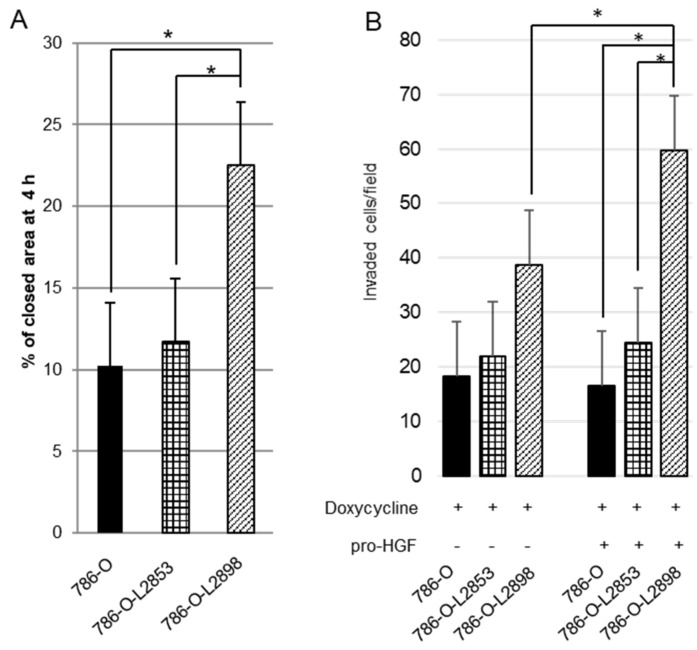
Effect of HAI-2 knockdown or engineered expression on motility and invasiveness of 786-O cells. Cell motility was evaluated by wound healing assay (**A**), and invasion assay (**B**) through Matigel with and without endogenous pro-HGF. Values are means ± standard deviation of triplicate experiments. * *p* < 0.05, Mann–Whitney *U* test.

As shown in Figure 4, doxycycline-induced HAI-2 knockdown and engineered expression were confirmed by RT-qPCR. Samples of mRNA were extracted from 786-O-Luc2, HAI-2 stable knockdown 786-O-L2898, and HAI-2 overexpressed 786-O-L2853 cells cultured under condition of 0.5 µg/mL of doxycycline. Apparent downregulation of HAI-2 in 786-O-L2898 cells and engineered expression of HAI-2 in 786-O-L2853 was confirmed (Figure 4). Of interest, the expression of matriptase in 786-O-L2898 cells was upregulated (Figure 4A), whereas downregulation was observed in 786-O-L2853 cells (Figure 4B). No significant difference in cell growth was observed in any cell line; however, significant upregulation of cell motility was observed in 786-O-L2898 cells by wound healing assay compared with 786-O-Luc2 cells and 786-O-L2853 cells (Figure 5A).

We then performed invasion assay using Matrigel. As a result, invasive activity was more aggressive in 786-O-L2898 cells compared with parental 786-O-Luc2 or 786-O-L2853 cells (Figure 4A). Furthermore, invasiveness was more upregulated in the presence of pro-HGF, suggesting the significance of ligand-dependent MET activation in the cells.

Next, we examined the expression of 84 cancer metastasis-related molecules using the RT^2^ Profiler PCR Array system. As a result, significant upregulation of chemokine ligand 7 (CCL7) and chemokine receptor 4 (CXCR4) in 786-O-L2898 was observed (Table 1). On the other hand, CXCR4 and matrix metalloprotease 9 (MMP9) were downregulated in 786-O-L2853 cells. In addition, E-cadherin (cadherin 1) was slightly downregulated by knockdown of HAI-2. 

## 3. Discussion

High MET protein expression in bone metastasis of prostate cancer compared with primary site was reported by Knudsen et al. [24]. In breast cancer, the HGF/MET pathway activates the bone microenvironment through the Wnt-β-catenin pathway, and has an important role in the plasticity of bone metastasis [25]. We previously reported that higher expression of MET and matriptase was observed in RCC bone metastasis compared with the primary tumor [14]. In addition, inhibition of MET activation in cancer stem cells of RCC was reported to prevent bone metastasis [26]. The recent results of prespecified subgroup analyses in the METEOR trial revealed significantly improved median overall survival in RCC patients with bone metastasis treated by cabozantinib compared with everolimus [7]. This result lent considerable support to our hypothesis that the HGF/MET signaling axis plays an important role in RCC bone metastasis.

MET, receptor tyrosine kinase (RTK), is a disulfide-linked heterodimer consisting of an extracellular alpha chain (50 kDa) and a single-pass transmembrane β chain (145 kDa) [27,28]. The α chain, heterodimerized to the amino-terminal part of the β chain, forms a major HGF-binding site [27,28]. The intracellular segment consists of three components: namely a juxtamembrane domain which downregulates the kinase activity of MET following phosphorylation of Ser975; a catalytic site that is a so-called activation loop that positively actuates the kinase activity of MET following phosphorylation of Tyr1234 and Tyr1235; and a carboxy-terminal multifunctional docking site composed of Tyr1349 and Tyr1356, which leads to downstream signaling through several intracellular adaptor proteins [15,27,28]. In human cancer, three different means of MET activation have been reported: HGF-dependent (autocrine or paracrine system); overexpression of MET (oligomerization of MET causes reciprocal activation); and activating point mutations (such as hereditary papillary renal cell carcinoma). The latter two of these are ligand independent [11,14,15,27,28]. In a minority of cancers, the *MET* gene is reported to act as a driver oncogene; however, the majority of cancers utilize wild-type MET, which relies on HGF, as an expedient to confer a malignant phenotype [12,16].

Activation of MET can lead to the activation of several major intracellular signaling pathways. In cancer cells, activation of these signaling pathways is reported to promote cell proliferation, survival (anti-apoptosis), motility, invasiveness, drug resistance, the maintenance of the cancer stem cell-like phenotype, and epithelial-mesenchymal transition (EMT) [27,28]. High MET expression with worsening prognosis has been reported in a large number and variety of human cancers, including RCC [11,14,27,28]. 

The *HAI-2* gene is encoded by *SPINT2*, which is located on chromosome 19q13.2 [12,29]. HAI-2 was initially identified in culture medium of a human gastric cancer cell line (MKN45), and independently purified from placenta as placental bikunin [12,30,31]. The protein has two extracellular Kunitz-type serine protease inhibitor domains, and sufficient inhibitory activity towards all pro-HGF-activating enzymes has been reported [12]. Under physiological conditions, HAI-2 is expressed in various epithelial cells, and the subcellular localization is reported to have been observed in cytoplasm by immunohistochemistry [12]. The function is also reported to maintain the integrity of intestinal epithelium through regulation of matriptase activity and epithelial cell adhesion molecule (EpCAM) turnover [32]. Downregulation of the *SPINT2* gene by hypermethylation is reported in cancers, including glioblastoma, hepatoma, melanoma, and RCC [12]. Reversion of HAI-2 in these cancer cells revealed suppression of invasive growth. This study revealed that that the expression of HGF and matriptase in bone metastasis of intra-cardiac mouse model increased, while that of HAI-2 decreased. Furthermore, phosphorylation of MET was also enhanced in bone metastasis. This result indicated that bone microenvironment influenced a decrease in the expression of HAI-2, leading to upregulation of the HGF/MET signaling axis. Indeed, stable knockdown of HAI-2 (786-O-L2898) revealed upregulated invasive activity and motility, and invasive capability was enhanced in an HGF-dependent manner. In the future, we would like to analyze the function of HAI-2 in the system in vivo using 786-O-L2853 and 786-O-L2898 cells.

In lung cancer, amplification of *MET* is reported to be a mechanism of resistance to epidermal growth factor receptor-targeted tyrosine kinase inhibitors (EGFR-TKIs) [33]. On the other hand, overexpression of HGF was identified in 61% of lung cancer patients with resistance to EGFR-TKIs overlapping *MET* amplification [33]. In addition, expression of HGF is reported to be significantly increased in patients with acquired resistance to EGFR-TKIs compared with pretreatment group [33]. Analysis of resistance to anti-MET therapy also revealed the significance of HGF-promoted resistance in drug potency and efficacy [34]. Furthermore, phosphorylation of MET with auto-activating mutation was significantly enhanced by HGF stimulation compared with the absence of HGF [35]. These reports indicated the importance of activated-HGF in the MET signaling axis [33,34,35]. Because pro-HGF is frequently expressed in various human cancers and abundantly expressed by cancer associated stromal cells, pro-HGF activation systems are also major targets in anti-MET treatment. In RCC, downregulation of HAI-2 by hypermethylation of the promoter lesion has been reported in several studies [11,12]. Indeed, the majority of RCC cell lines in our study revealed no expression of HAI-2, and 786-O cells expressing HAI-2 showed downregulation of HAI-2 in bone metastasis. Therefore, HAI-2, a sufficient multi-inhibitor of pro-HGF-activating proteases, is a potential candidate as a novel HGF/MET-targeted agent.

Results of PCR array revealed the reciprocal expression of CCL7, CXCR4, and MMP9 with HAI-2, whereas Cadherin 1 expressed non-reciprocally with HAI-2. CCL7 and CXCR4 are well known chemokines that promote cancer progression [30,31]. MMP9 may correlate with increased invasive activity in HAI-2 knockdown 786-O cells through degradation of extracellular matrix [36]. In addition, downregulation of cadherin 1 (E-cadherin) in 786-O-L2898 may also suggest the process of EMT. Morphological appearance in bone metastasis (Figure 3A,D) may also support this. However, further examination is required to clarify the correlation between HAI-2 and these molecules.

## 4. Materials and Methods

### 4.1. Cell Culture

Renal cell carcinoma cell lines (Caki-1, Caki-2, A498) and the Hela cell line were purchased from American Type Culture Collection (Manassas, VA, USA). MRT-1, a renal cell carcinoma cell line, was established in our laboratory [37]. The RBM1-IT4 cell line, which is an aggressive subline of bone metastasis-derived RCC cell line RBM1, was provided by T. Karashima (Kochi University, Kochi, Japan) under approval of K. Weber (Penn Medicine, PA, USA). The Luciferase transfected 786-O cell line (786-O-Luc2) was provided by N. Shibasaki (Kyoto University, Kyoto, Japan). All cells were cultured in low-glucose Dulbecco’s Modified Eagle Medium (DMEM, Gibco, Carlsbad, CA, USA) with 10% fetal bovine serum (FBS) (Gibco), 1% Pen/Strep at 37 degrees Celsius in a humidified atmosphere of 5% CO_2_.

### 4.2. Animal Experiments

All experiments involving laboratory animals were performed in accordance with the Guidelines for Animal Experiments of Miyazaki University (Permit Number: 2013-536, 11 December 2013). Female athymic nude (BALB/AJcl-nu/nu) mice (Charles River Laboratories Japan, Yokohama-shi, Japan) were maintained under germ-free conditions until 5 weeks old.

For intra-cardiac injection, 786-O-Luc2 cells were cultured and then detached by trypsin EDTA at 70–80% confluent before resuspension in PBS to a final concentration of 1 × 10^6^/100 µL. Mice were anesthetized with 0.15 mL of 5-fold diluted somnopentyl administered 1–2 mm to the left side of the sternal midline, and 100 µL of cell suspension was injected using a 28G needle. As a control, 1 × 10^5^ cells/10 µL of 786-O parental cells were injected subcutaneously into nude mice. After injection to left cardiac ventricle, whole-body bioluminescent imaging was performed to confirm the success of the procedure. At 6 weeks following injection, whole-body bioluminescent imaging was conducted to confirm the formation of bone metastasis. Mice with bone metastasis were then sacrificed for tissue extraction. Mice with subcutaneous implantation were also sacrificed and the implanted tissue was extracted at same time.

### 4.3. RNA Extraction and RT-PCR

Tissue was homogenized by beads crusher (µT-1) before extracting RNA. Total RNA was extracted using PureLink RNA Mini Kit (Thermo Fisher SCIENTIFIC, Waltham, MA, USA) according to the manufacturer’s directions. Genes of interest were amplified from 2 mg DNase I-treated total RNAs using Thunderbird Reverse Transcriptase (Toyobo, Tokyo, Japan) and random primers. 

### 4.4. Real-Time Quantitative PCR

Real-time RT-PCR analyses were performed with a Thermal Cycler Dice Real-Time System II (Takara, Shiga, Japan). Reaction mixture (25 µL) containing 2 µL of cDNA template, 1 µL each of sense and anti-sense primers, and 1 × SYBR Premix Ex Taq II (Takara, Shiga, Japan) were amplified as follows: hold at 95 °C for 30 s and 40 cycles at 95 °C for 5 s, 60 °C for 30 s, and final dissociation stage (95 °C for 15 s, 60 °C for 30 s, and 95 °C for 15 s). Glyceraldehyde-3-phosphate dehydrogenase (GAPDH ) was used as an internal control. The results were evaluated using the Thermal Cycler Dice Real Time System software program (Takara Bio, Shiga, Japan). The delta-delta Ct (ddCt) algorithm [38] was used to analyze the relative changes in gene expression. The primers were as follows: GAPDH forward, 5′-GCACCGTCAAGGCTGAGAAC-3′ and reverse, 5′-TGGTGAAGACGCCAGTGGA-3′; HAI-1 forward, 5′-GGTGACACGGATGTCAGGGTA-3′ and reverse, 5′-CACTGTCAGCTGGAACAGGTAGG-3′; HAI-2 forward, 5′-GACGGAAACAGCAATAATTACCTGA-3′ and reverse, 5′-TTGAACATATCGCTGGAGTGGTC-3′; matriptase forward, 5′-GAGCAAGGGCAACCCTGAGT-3′ and reverse, 5′-CCCAACAACACGAGCCTGTC-3′; HGF forward, 5′-GTTCAATGTGGGACAAGAACATGG-3′ and reverse, 5′-GGATTTCGGCAGTAATTCTCATTCA-3′; MET forward, 5′-TCCCATCAACAGGACTACACACTT-3′ and reverse, 5′-GCTGCAGGTATAGGCAGTGACAA-3′.

### 4.5. Immunohistochemistry

Formalin-fixed paraffin-embedded sections were prepared according to routine method. Specimens of bone metastasis were subjected to decalcification using 10% ethylenediamine–tetra-acetic acid (pH 7.2) for 12–24 h for use in hematoxylin, eosin stain, and immunohistochemistry. For immunohistochemistry, sections were processed for antigen retrieval (microwave in 10 mM citrate buffer, pH 6.0 for 10 min), followed by treatment with 3% H_2_O_2_ in methanol for 10 min and washed in tris-buffered saline (TBS) twice. After blocking in 3% bovine serum albumin and 5% goat serum in phosphate buffered saline for 2 h at room temperature, the sections were incubated with primary antibodies overnight at 4 °C. Anti-human MET rabbit polyclonal antibody was purchased from Immuno-Biological Laboratories (IBL, Gunma, Japan), anti-human phosphor-MET (Tyr1235) rabbit polyclonal antibody was also purchased from IBL. Negative controls did not include the primary antibody. Sections were then washed in TBS and incubated with Envision-labeled polymer reagent (DAKO, Santa Clara, CA, USA) for 30 min at room temperature. Sections were exposed to nickel, cobalt-3, 3-diaminobenzidine (Immunopure Metal Enhanced DAB Substrate Kit; Piece, Rockford, IL, USA), and counterstained with hematoxylin.

### 4.6. Construction of Lentiviral Vectors and Viral Transduction into 786-O-Luc2 Cells to Produce Doxycycline-Induced HAI-2 Stable Knockdown and Overexpression Cells

The most efficient target sequence for RNA interference of HAI-2 was selected (SPINT2; HSS173811, Invitrogen (Carlsbad, CA, USA). On the other hand, the whole coding region of HAI-2 was also constructed by PCR using full-length HAI-2 cDNA as a template. Each cDNA was subcloned into the tetracycline (TET)-inducible vector pCLVi(3G)-Puro (SIRON Biotech, Martinsried, Germany) generating a lentiviral expression vector pCLVi(3G)-shmir-HAI-2-Puro or pCLVi(3G)-HAI-2-Puro. HAI-2-lentiviral particles were used for transduction at a multiplicity of infection (MOI) of 2.5 for 72 h. Puromycin resistance was used as a marker gene at the concentration of 1.5 µg/mL for 20 days and puromycin resistance cells were selected. Then, we prepared HAI-2 stable knockdown 786-O-Luc2 cell line (786-O-L2898) and HAI-2 stably overexpressed 786-O-Luc2 cell line (786-O-L2853). For the confirmation of RNA interference and overexpression, doxycycline was added at a concentration of 0.5 µg/mL and cultivated. After 48 h, total RNA was extracted for RT-qPCR. 

### 4.7. Wound Healing Assay

786-O, 786-O-L2898, and 786-O-L2853 cells were cultured with 5 µg/mL of doxycycline 48 h prior to assay. Cells were harvested at 80% confluency and re-suspended at 5 × 10^5^ cells/mL. 500 µL of cell suspension was added to a 24-well plate, into which a wound field insert was placed (CytoSelect 24-Well Wound Healing Assay, Cell Biolabs, Inc., San Diego, CA, USA). After 24-h incubation, the wound field insert was carefully removed and the wound field area was measured at start and 4 h. The percent closure was determined as follows: percent closure (%) = migrated cell surface area/total surface area × 100. The closure rate of each cell line was compared using the Mann–Whitney *U* test.

### 4.8. Invasion Assay

Invasion assay was performed following the protocol for 24-well Corning BioCoat Matrigel Invasion Chamber (Corning, MA, USA). 786-O-Luc2 cells (parent), 786-O-L2898, and 786-O-L2853 cells were maintained in DMEM with 0.1% bovine serum albumin (BSA, Roche, Penzberg, Germany) for use in invasion assay. Each cell line was cultured with 0.5 µg/mL of doxycycline 48 h prior to assay. Cells were harvested and re-suspended in DMEM with 0.1% BSA at 1.0 × 10^5^ cells/mL, and then 0.5 × 10^5^ cells/500 µL were seeded in each well. After 24-h incubation with and without recombinant pro-HGF (final concentration in culture 40 ng/mL), invaded cells were counted and compared using the Mann–Whitney *U* test. Preparation of pro-HGF has been described in previous reports [11,21]. 

### 4.9. PCR Array

RT^2^ Profiler PCR Array system (QIAGEN, Cat. No. PAHS-028Z, Hilden, Germany) was used to determine the molecules affected by HAI-2 expression, which promotes 786-O cell motility. 786-O, 786-O-L2898, and 786-O-L2853 cells which were exposed to 0.5 µL/mL of doxycycline were used for the assay. Total RNA was extracted from each cell line and cDNA was synthesized using RT^2^ First Standard Kit (QUIAGEN). cDNA from each cell line was mixed with RT^2^ SYBR^®^ Green qPCR Master Mix (QIAGEN, Cat. No. 330529) and H_2_O according to the manufacturer’s protocol. Subsequently, 25 μL of the mixture was placed into each well of the PCR array (96-well plate). Forty cycles of three-step programs (95 °C for 15 s, 55 °C for 30 s, and 72 °C for 30 s) were performed after 10 min at 95 °C. Values were obtained for the threshold cycle (Ct) for each gene and normalized using the average of housekeeping genes of *GAPDH* and *ACTB*. Changes of mRNA expression were compared between each cell line and the results were reported as fold change: 2-fold or greater change was considered as significant change.

### 4.10. Statistics

Statistical analysis was performed using SPSS statics, version 22 (SPSS, Chicago, IL, USA). Significance was set at *p* < 0.05.

## 5. Conclusions

We employed a mouse model of human RCC bone metastasis, and identified downregulation of HAI-2 and increased phosphorylation of MET with upregulation of HGF and matriptase in bone metastasis, suggesting enhanced ligand-dependent MET activation in an autocrine manner. In vitro examination revealed knockdown of HAI-2-induced upregulation of cell motility and invasiveness in 786-O cells. Furthermore, invasive activity was more enhanced in the presence of pro-HGF. These data suggested the significance of ligand-dependent MET activation in RCC bone metastasis, and that HAI-2 may an important regulator in this system.

## Figures and Tables

**Table 1:** Expression of molecules related to cancer metastasis in 786-O-L2898 and 786-O-L2853 cells, identified by RT^2^ Profiler PCR Array system.

Gene Symbol	Gene Name	786-O-L2898 Fold Change	786-O-L2853 Fold Change
CCL7	Chemokine (C-C motif) ligand 7	4.29	1.33
CDH1	Cadherin 1, type 1, E-cadherin	−1.08	1.72
CXCR4	Chemokine (C-X-C motif) receptor 4	2.57	−2.45
MMP9	Matrix metalloprotease 9	1.69	−7.29
